# Comparative Analysis of Polyphenol-Rich Extracts from *Hamamelis virginiana* Leaves and Bark: ROS Scavenging and Anti-Inflammatory Effects on Skin Cells

**DOI:** 10.3390/molecules30173572

**Published:** 2025-08-31

**Authors:** Magdalena Wójciak, Wiktoria Pacuła, Katarzyna Tyszczuk-Rotko, Aleksandra Ziemlewska, Martyna Zagórska-Dziok, Zofia Nizioł-Łukaszewska, Rafał Patryn, Anna Pacian, Ireneusz Sowa

**Affiliations:** 1 Department of Analytical Chemistry, Medical University of Lublin, Chodźki 4a, 20-093 Lublin, Poland; wiktoria.pacula@umlub.edu.pl; 2 Faculty of Chemistry, Institute of Chemical Sciences, Maria Curie-Skłodowska University in Lublin, 20-031 Lublin, Poland; katarzyna.tyszczuk-rotko@mail.umcs.pl; 3 Department of Technology of Cosmetic and Pharmaceutical Products, Medical College, University of Information Technology and Management in Rzeszow, Sucharskiego 2, 35-225 Rzeszow, Poland; aziemlewska@wsiz.edu.pl (A.Z.); mzagorska@wsiz.edu.pl (M.Z.-D.); zniziol@wsiz.edu.pl (Z.N.-Ł.); 4 Department of Humanities and Social Medicine, Medical University of Lublin, Chodźki 7, 20-093 Lublin, Poland; rafal.patryn@umlub.edu.pl; 5 Department of Health Education, Medical University of Lublin, Staszica 4/102, 20-081 Lublin, Poland; anna.pacian@umlub.edu.pl

**Keywords:** witch hazel, anti-inflammatory, oxidative stress, antioxidant enzymes, metalloproteinases, polyphenols

## Abstract

*Hamamelis virginiana* (witch hazel) is traditionally used in dermatology for its antibacterial and anti-inflammatory effects. However, the number of studies on its chemical composition and potentials in skin protection remains limited. This study aimed to investigate the qualitative and quantitative composition of polyphenolic compounds in the leaves and bark of the plant, as well as to explore their antioxidant, anti-inflammatory, and extracellular matrix (ECM)-protective activities in skin-relevant cell models. Human dermal fibroblasts and keratinocytes were exposed to oxidative and inflammatory stimuli and pretreated with leaf and bark extracts. ROS levels, antioxidant enzyme activity (SOD, GPx, CAT), pro-inflammatory cytokines (IL-6, IL-1β, TNF-α), and inhibition of collagenase, hyaluronidase, and elastase were assessed. Both extracts strongly reduced ROS levels, enhanced SOD activity, and significantly decreased pro-inflammatory cytokines. Bark extract also exhibited potent inhibitory activity against collagenase and elastase. UPLC-DAD-MS analysis revealed that both plant parts contained high levels of tannins; however, the leaf extract showed a more diverse composition, including more complex tannin forms and a significant amount of flavonoids from the quercetin and kaempferol class. In conclusion, *H. virginiana* leaf and bark extracts demonstrate multifunctional antioxidant and anti-inflammatory properties, supporting their potential use in cosmeceuticals and dermatological formulations targeting skin aging and inflammation.

## 1. Introduction

**Figure 1:**
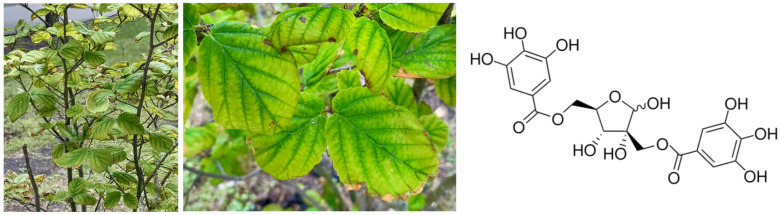
Photograph of *Hamamelis virginiana* (witch hazel) in its natural habitat with a magnified view of the leaves and the chemical structure of hamamelitannin—its major constituent.

Plants rich in polyphenols are of great importance in skincare and cosmetology due to their strong antibacterial, antioxidant, anti-inflammatory, and protective properties [1]. Polyphenols are known to help neutralize free radicals, reduce oxidative stress, and prevent premature skin aging caused by environmental factors such as UV radiation and pollution. They also support skin regeneration and improve its overall resilience and appearance [2]. One such polyphenol-rich plant is *Hamamelis virginiana* L. (witch hazel), a plant species from the Hamamelidaceae family (Figure 1), widely recognized for its therapeutic potential in both traditional medicine and modern dermatology [3]. Native to North America, particularly the eastern regions of the United States and southern Canada, *H. virginiana* has been historically used for the treatment of various skin-related conditions such as inflammation, wounds, and irritation caused by insect bites or poison ivy [4]. Over the centuries, these traditional applications have been adopted and expanded across Europe and other regions, where witch hazel extracts are now commonly used in both herbal medicine and cosmetic formulations [3,5].

From the perspective of current legal regulations, both those of the European Union (EU) and national laws, including Polish legislation, *H. virginiana* can be used in cosmetology, medicinal products, and dietary supplements, with its most widespread application found in cosmetic formulations. According to EU law, a cosmetic product is defined as “any substance or mixture intended to be applied to the external parts of the human body (epidermis, hair, nails, lips, external genital organs) or to the teeth and oral mucosa, with the exclusive or principal purpose of cleaning, perfuming, changing their appearance, protecting, keeping them in good condition, or correcting body odour” [6,7]. Depending on the intended use, different legal frameworks apply, including pharmaceutical, food, and cosmetic product regulations. These acts define, among others, product standardization requirements including composition and concentration, safety, and rules governing marketing and sale. Therefore, phytochemical and biological studies of plant raw materials, which form the basis of active cosmetic ingredients, are of particular importance.

The therapeutic properties of *H. virginiana* are primarily attributed to tannins, which are high-molecular-weight polyphenolic compounds They are present in both the bark and leaves, with contents of approximately 8–12% and 3–10%, respectively. Tannins are generally classified into two main groups: condensed tannins (proanthocyanidins) and hydrolyzable tannins. Condensed tannins are composed of flavan-3-ol units, such as catechin, epicatechin, gallocatechin, or epigallocatechin. These monomeric units are linked by carbon–carbon bonds, typically between the C4 position of one unit and the C6 or C8 position of the next, making them resistant to hydrolysis [8,9]. The degree of polymerization can vary, leading to oligomers or polymers. In contrast, hydrolyzable tannins are polyphenolic compounds with a central sugar molecule (commonly glucose) esterified by phenolic acids. These ester bonds can be relatively easily hydrolyzed by acids, bases, or specific enzymes, which is why they are called “hydrolyzable” tannins. The most common types of phenolic acids involved are gallic acid (forming gallotannins) and ellagic acid (forming ellagitannins). Structurally, gallotannins are made up of several gallic acid units attached to glucose, while ellagitannins form when some gallic acid units are oxidatively coupled to produce hexahydroxydiphenic acid, which spontaneously converts to ellagic acid upon hydrolysis [8,9]. Tannins exhibit significant biological activity relevant to the treatment of skin disorders. Due to their astringent, anti-inflammatory, and antimicrobial properties, tannins can help reduce skin irritation, limit microbial growth, and promote wound healing [9]. For example, it has been found that hamamelitannin (Figure 1), a specific compound found in *H. virginiana*, exhibits strong free radical scavenging activity and the ability to protect from oxidative stress and UVB-induced cell damage [10,11,12]. It also possesses notable antimicrobial and antibiofilm activity, particularly against drug-resistant *Staphylococcus aureus* and *S. epidermidis* [13], and has demonstrated significant anti-inflammatory properties [14,15].

Despite the long history of the use of *H. virginiana* in dermatological applications, modern studies exploring its biological potential remain limited, with most focusing primarily on its antibacterial properties. Moreover, phytochemical data on the polyphenolic composition of *H. virginiana* raw materials are still insufficient; although a few studies provide qualitative data based on mass spectrometry, quantitative data are lacking, or the authors have primarily focused on the extraction of tannin-related components [16,17]. Therefore, the aim of this study was to provide insight into the content of low-molecular-weight polyphenolic compounds in the leaves and bark of *H. virginiana*, using various extraction methods. Furthermore, this study aimed to evaluate the effects of polyphenol-rich extract from witch hazel leaf and bark on skin cells, with a focus on cellular responses to oxidative stress, including their reactive oxygen species (ROS) scavenging capacity, influence on the antioxidant enzyme system, and anti-inflammatory action. By employing in vitro models and advanced analytical techniques, this research seeks to deepen our understanding of the biological potential of *H. virginiana* in the context of skin protection.

## 2. Results

### 2.1. Phytochemical Investigations

#### 2.1.1. UPLC-(ESI)MS-DAD Profiling

**Figure 2:**
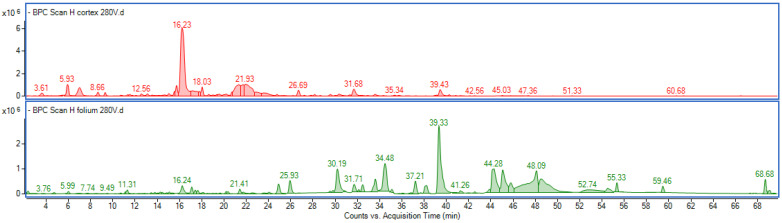
Representative base peak chromatograms (BPCs) obtained in negative ion mode using an ionization energy of 280 V for *H. virginiana* bark extract (red) and leaf extract (green).

Components of the extracts were identified based on high-resolution mass spectrometry data obtained using different ionization energies, which allowed the monitoring of characteristic fragment ions. The UV–VIS spectra were recorded in the range of 200–600 nm and used to classify the components into specific classes, including gallic acid derivatives (UV–VIS spectra showing maximum absorbance at ~270 nm), flavan-3-ols such as catechin and epicatechin (max at ~220 nm and ~280 nm), flavonoids including quercetin and kaempferol derivatives (characteristic maxima at ~255 and 350–365 nm), and ellagic acid derivatives (showing typical maxima at ~254 and 365 nm) [18,19,20]. Gallic acid derivatives exhibited two characteristic fragment ions at *m*/*z* = 169 and 125 ([M − H]^−^), corresponding to deprotonated gallic acid and its decarboxylation product, respectively. Catechin and epicatechin derivatives showed a common fragment ion at *m*/*z* = 289 corresponding to [M − H]^−^. Quercetin derivatives were identified by a diagnostic ion at *m*/*z* = 300, while kaempferol derivatives showed a diagnostic ion at *m*/*z* = 284, both ions corresponding to [M − H]^−^. An example of chromatograms obtained for the bark and leaf extracts is shown in Figure 2. Representative UV_VIS and MS spectra of several specific components characteristic of particular classes of metabolites are provided in the Supplementary Materials (Figure S1).

Molecular formulas were generated using MassHunter software (ver. 10.0), with the difference between theoretical and observed mass not exceeding 5 ppm. The identities were further confirmed using authentic standards and relevant literature, including the studies of Duckstein et al. and Burico et al., who reported detailed mass spectrometry-based profiles for leaf water extracts [16,17]. The chromatographic and mass spectrometry data are presented in Table 1.

In general, the qualitative tannin profile was similar for both parts of the plant. However, the leaf extract contained more complex compounds, such as octa-O-galloylhexose (*m*/*z* = 1395.144) and nona-O-galloylhexose (*m*/*z* = 1547.156), which were not found in the bark. Moreover, bark extract lacked digallic acids (*m*/*z* = 321.024) and p-coumaroylquinic acids (*m*/*z* = 337.094). In addition, the leaf extract was abundant in flavonoid compounds, and the flavonoid profile was similar to those reported by Duckstein et al. [17]. However, based on comparison with standards, we additionally identified several quercetin and kaempferol derivatives. Extracted ion chromatograms for different flavonoid derivatives are shown in Figure S2.

#### 2.1.2. Quantitative Results

As the extraction method has a significant impact on the final phytochemical profile, three different techniques were employed to investigate the polyphenolic composition of *H. virginiana*, including ultrasound-assisted extraction (UAE), heat reflux extraction (HRE), and accelerated solvent extraction (ASE). Each method offers distinct advantages in terms of extraction efficiency, selectivity, and suitability for thermolabile compounds. The test was carried out in different temperatures. Examples of chromatograms for different extraction methods are shown in Figures S3 and S4. The results of quantification of the main metabolites in *H. virginiana* leaf and bark extracts, using conditions that ensured the highest extraction yield, are summarized in Table 2 and Table 3, respectively.

Overall, bark extracts contained higher concentrations of several characteristic polyphenols including catechin, hamamelitannin, galloylquinic acids, and galloyl hexoses compared with leaf extracts. Particularly notable was the content of hamamelitannin, which reached 62.75 mg/g in the bark extract (ASE), more than 260 times higher than in the leaf extract (0.275 mg/g). The total tannin content in the bark accounted for approximately 9% of the raw material’s dry weight, with hamamelitannin alone representing over 70% of that fraction. In contrast, leaf extracts were characterized by a broad spectrum of flavonol glycosides (quercetin and kaempferol derivatives), which were absent in the bark. The total amount of quercetin derivatives was 1.09 mg/g (ASE), and kaempferol derivatives reached 1.85 mg/g (ASE). The leaf extract also contained high amounts of phenolic acids, such as ellagic acid (ninefold higher than in bark), and protocatechuic and gallic acids (2-fold higher). Chlorogenic acid was found only at trace levels in the bark, while in the leaf extract, it was present at 0.36 mg/g.

### 2.2. Biological Assays

Based on the obtained results, the ASE technique was used to prepare ethanolic extracts from *H. virginiana* leaves and bark for biological assays. The extracts were freeze-dried and chemically characterized, with the quantity of compounds calculated per gram of dried extract. The detailed qualitative profile of phenolic compounds, expressed as the sum of specific isomeric forms, is summarized in Table S1. The bark extract was dominated by hamamelitannin (243.6 mg/g), catechin/epicatechin (24.68 mg/g), and various galloylated derivatives, including galloyl-hexoses (43.78 mg/g) and di- to hepta-O-galloylhexosides (39.03 mg/g). In contrast, the leaf extract contained a more diverse range of compounds with lower amount of tannin-related compounds (206.4 mg/g vs. 386.5 mg/g in bark extract) but a significantly higher presence of flavonoids, particularly kaempferol and quercetin derivatives (total 14.03 mg/g), as well as a notable amount of ellagic acid (21.52 mg/g).

#### 2.2.1. Cytotoxicity Analysis

The impact of the tested samples on cell viability was assessed using two tests: Neutral Red and Alamar Blue. The first one assesses cell health based on the ability of lysosomes to uptake the dye, reflecting the integrity of cell membranes and the proper function of these organelles. The Alamar Blue test assesses metabolic activity, indicating the efficient functioning of mitochondria and the enzymatic activity of cells [21].

Cells were exposed to the extracts obtained from witch hazel bark and leaves at various concentrations: 50, 125, and 250 µg/mL. This study included two types of skin cells: keratinocytes (HaCaT line), representing the epidermis, and human fibroblasts (HDFs), responsible for dermal functions. The results obtained allowed for the assessment of the cytotoxicity of the tested substances and their impact on cell metabolism and the integrity of cellular structures.

**Figure 3:**
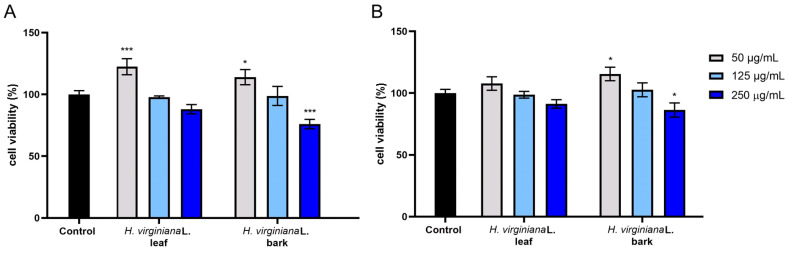
The reduction in resazurin (**A**) and Neutral Red dye uptake (**B**) in cultured keratinocytes (HaCaTs) after 24 h of exposure to *H. virginiana* leaf and bark extracts. The results are presented as the mean ± standard deviation (SD) from three independent experiments, each performed in triplicate. *** *p* < 0.001, * *p* = 0.0481.

In the Alamar Blue and Neutral Red assays performed with HaCaT cells, a strong concentration-dependent effect on cell viability was observed, with a tendency toward decreased viability at higher concentrations. However, at the lowest concentration, all samples increased cell viability relative to the control, with values of approximately 110–120%. In the Alamar Blue assay, a clear cytotoxic effect at the highest concentration was observed for the witch hazel bark extract (Figure 3).

**Figure 4:**
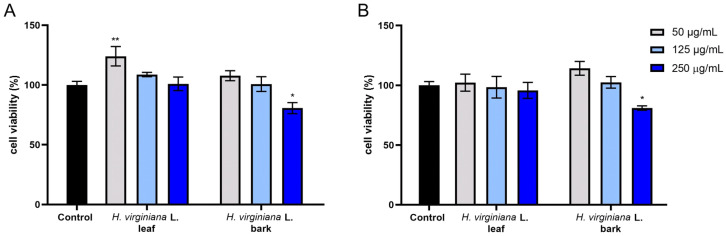
The reduction in resazurin (**A**) and Neutral Red dye uptake (**B**) in cultured fibroblasts (HDFs) after 24 h of exposure to *Hamamelis virginiana* L. leaf and bark extracts. The results are presented as the mean ± standard deviation (SD) from three independent experiments, each performed in triplicate. ** *p* = 0.0074, * *p* < 0.05.

In the Alamar Blue and Neutral Red assays performed with HDF cells, the overall trends were very similar. Increased cell viability compared with the control was observed for the bark extract in Neutral Red and for the leaf extract in Alamar Blue, reaching approximately 120–125%. With increasing concentrations, cell viability decreased. At the highest concentration of the bark extract, a clear cytotoxic effect was observed in both assays, with values falling clearly below the control (Figure 4).

#### 2.2.2. Intracellular ROS Levels in Skin Cells

**Figure 5:**
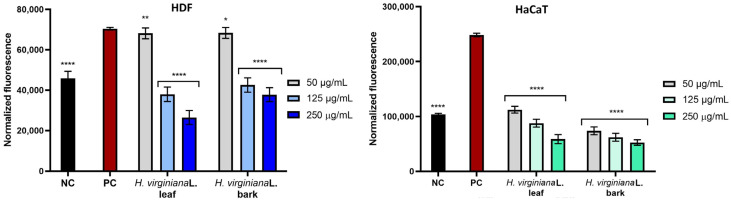
The effect of *H. virginiana* leaf and bark extracts on the intracellular level of reactive oxygen species (ROS) in human keratinocytes (HaCaTs) and in human fibroblasts (HDF cells) exposed on H_2_O_2_. ROS levels were determined using the H_2_DCFDA fluorescent probe. The negative control (NC) consisted of cells untreated with either extracts or H_2_O_2_, while the positive control (PC) consisted of cells exposed to 500 μM H_2_O_2_. Data are presented as mean ± SD from three independent experiments, with each sample tested in triplicate. **** *p* < 0.0001, ** *p* = 0.0054, * *p* = 0.0116.

The intracellular accumulation of reactive oxygen species (ROS) was evaluated in human keratinocytes (HaCaTs) and dermal fibroblasts (HDFs) following treatment with leaf and bark extracts of *H. virginiana* under oxidative stress conditions induced by 500 μM hydrogen peroxide (H_2_O_2_). As shown in Figure 5, exposure to H_2_O_2_ caused a substantial increase in ROS levels in both cell types. Notably, the ROS elevation was more pronounced in HaCaT cells compared with HDFs, suggesting a higher sensitivity of keratinocytes to oxidative stress. This observation may reflect cell type-specific differences in antioxidant defense mechanisms or susceptibility to redox imbalance. In contrast, untreated control cells, which were not exposed to H_2_O_2_ or *H. virginiana* extracts, exhibited low baseline levels of ROS, consistent with physiological redox homeostasis. Pre-treatment with *H. virginiana* extracts significantly reduced the H_2_O_2_-induced ROS accumulation in a concentration-dependent manner. Both extracts exhibited a significant ability to reduce intracellular ROS levels across the entire tested concentration range (50–250 μg/mL) in both HaCaT and HDF cells. Importantly, both leaf and bark extracts (at concentrations ranging from 125 to 250 μg/mL) demonstrated a marked ability to reduce ROS levels not only in comparison with H_2_O_2_-treated cells but also below those observed in untreated controls. This suggests that the extracts possess intrinsic antioxidant activity, with the effect of displacing the cellular redox state towards a less oxidative baseline. It was also observed that *H. virginiana* bark extracts possess higher intrinsic antioxidant activity in HaCaT compared with the leaf extract. Overall, these findings demonstrate that *H. virginiana* extracts exert strong antioxidative effects in skin cells exposed to oxidative stress. Their ability to reduce intracellular ROS levels even below physiological norms highlights their potential for dermatological applications focused on redox balance restoration and protection against oxidative damage.

#### 2.2.3. Effect on Antioxidant Enzymes

**Figure 6:**
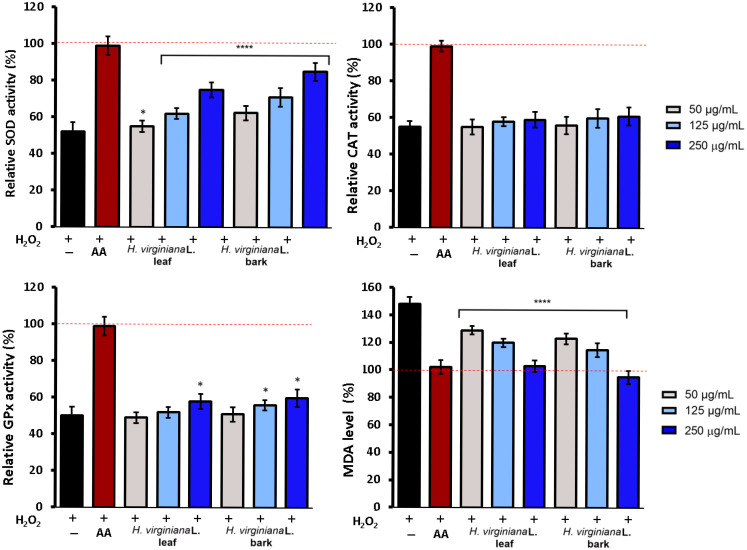
Effect of co-treatment human dermal fibroblasts (HDFs) with H_2_O_2_ and different concentrations of *H. virginiana* leaf and bark extracts on antioxidant enzyme activities, including superoxide dismutase (SOD), catalase (CAT), and glutathione peroxidase (GPx), as well as on malondialdehyde (MDA) levels. Values are expressed as percentages relative to the untreated control, indicated by the red line (100%). AA—ascorbic acid (positive control). * and **** indicate a statistically significant difference compared with H_2_O_2_-treated cells with *p* = 0.05 and *p* < 0.0001, respectively.

As the extracts demonstrated direct free radical scavenging activity, in the next stage, we investigated whether they also affect cellular antioxidant systems. The effect of co-treatment of human dermal fibroblasts (HDFs) with H_2_O_2_, used as an oxidative stress inducer, and the extracts on superoxide dismutase (SOD) and catalase (CAT) activity is illustrated in Figure 6. The malondialdehyde (MDA) level was also assessed to evaluate the ability of the extracts to protect against lipid peroxidation. MDA is a product formed during the oxidative degradation of polyunsaturated fatty acids and is commonly used as a biomarker of oxidative stress and lipid membrane damage.

As can be seen, treatment with H_2_O_2_ activated antioxidant enzymes but subsequently reduced their activity as a result of depletion. Co-treatment with *H. virginiana* leaf and bark extracts partially restored SOD activity in a concentration-dependent manner, with the highest dose (250 µg/mL) showing the strongest effect, although it did not reach the level observed in the ascorbic acid (AA) control. Furthermore, the extracts had only a minor effect on GPx, whereas CAT activity remained largely unchanged in their presence. However, the H_2_O_2_-induced elevation of MDA was strongly attenuated by both leaf and bark extracts, with the highest concentrations reducing lipid peroxidation to the control level.

#### 2.2.4. Assessment of Anti-Inflammatory Activity

This study aimed to evaluate the levels of pro-inflammatory cytokines, including IL-6, IL-1β, and TNF-a, in human dermal fibroblast (HDF) cultures following treatment with selected extracts at a concentration of 50 and 125 µg/mL. These cytokines serve as critical indicators of inflammatory responses and are deeply involved in immune function and in pathological conditions such as skin aging, impaired wound healing, and chronic inflammation. Due to their significant roles, they are frequently used as biomarkers to assess cellular inflammatory activity, particularly in HDF fibroblasts, which contribute to skin regeneration and structural integrity. To simulate an inflammatory environment, the cells were stimulated with lipopolysaccharide (LPS). Cytokine activity was measured and presented as a fold of activity relative to a negative control group (untreated with either LPS or test substances) and then compared with a positive control group (treated with LPS alone).

**Figure 7:**
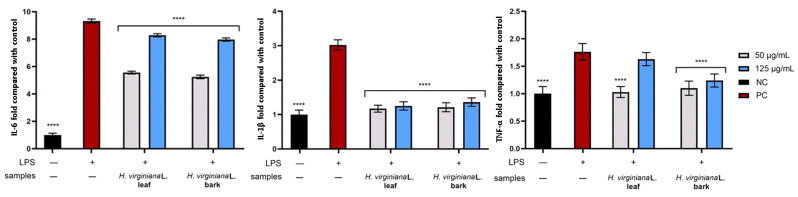
The effect of *H. virginiana* leaf and bark extracts after exposure to bacterial LPS (10 µg/mL) on the levels of interleukin 6 (IL-6), interleukin 1β (IL-1β), and tumor necrosis factor α (TNF-α) in human dermal fibroblasts (HDFs). Cytokine levels are expressed relative to the untreated control (NC). Statistical significance was calculated in comparison with LPS-treated cells (PC). Data are presented as mean ± SD from three independent experiments, with each sample tested in duplicate. **** *p* < 0.0001.

Figure 7 illustrates the effect of *H. virginiana* leaf and bark extracts on pro-inflammatory cytokine levels (IL-6, IL-1β, and TNF-α) in cells stimulated with bacterial LPS (10 μg/mL), expressed relative to the untreated control. LPS treatment significantly increased IL-6, IL-1β, and TNF-α levels compared with the control. Both leaf and bark extracts, at concentrations of 50 μg/mL and 125 μg/mL, markedly reduced IL-6 expression relative to LPS-treated cells. A similar trend was observed for IL-1β, where both extracts at both concentrations significantly lowered cytokine levels compared with LPS alone, approaching those of the control group. For TNF-α, a significant decrease in cytokine levels was also observed compared with LPS-stimulated cells, except for the leaf extract at 125 μg/mL. Interestingly, in the case of IL-6 and TNF-α, a greater reduction was observed at the lower concentrations of the tested extracts. Collectively, these results indicate that *H. virginiana* extracts, from both leaf and bark, effectively attenuate LPS-induced pro-inflammatory cytokine production, demonstrating strong anti-inflammatory potential.

#### 2.2.5. Assessment of Elastase, Hyaluronidase, and Collagenase Inhibitory Effect

Collagenase, elastase, and hyaluronidase are key enzymes responsible for breaking down key components of the extracellular matrix of collagen, elastin, and hyaluronic acid, leading to a decrease in skin elasticity, firmness, and hydration with age. Inhibiting their activity is considered an effective approach to anti-aging treatment. *H. virginiana* extract may help inhibit the activity of these enzymes, offering potential benefits in anti-aging skin care.

**Figure 8:**
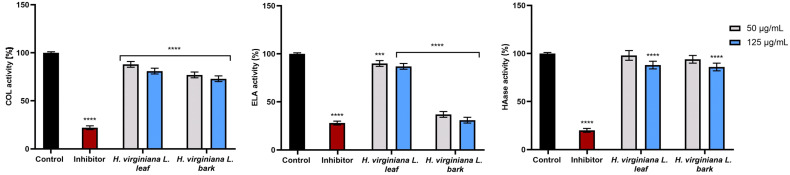
The effect of *H. virginiana* leaf and bark extracts on the activity of collagenase (COL), elastase (ELA), and hyaluronidase (HA) in human dermal fibroblasts (HDFs), expressed as a percentage of enzyme activity relative to untreated control cells. Data represent mean ± SD from three independent experiments, with each sample tested in duplicate. Succinyl-alanyl-alanyl-prolyl-valyl chloromethyl ketone (SPCK, 30 µM) was used as a positive control for elastase inhibition, 1,10-phenanthroline (300 µM) for collagenase inhibition, and tannic acid (300 µM) for hyaluronidase inhibition. **** *p* < 0.0001, *** *p* = 0.0006.

Figure 8 presents the effect of *H. virginiana* leaf and bark extracts on collagenase, elastase, and hyaluronidase activity. No statistically significant inhibition of hyaluronidase was observed. In the case of collagenase, a tendency toward enzyme inhibition was noted, but statistically significant inhibitory activity was demonstrated only for the bark extract at a concentration of 125 μg/mL. For elastase, no inhibition was detected with the leaf extract, whereas the bark extract exhibited a strong inhibitory effect at 125 μg/mL (0.730 ± 0.05-fold compared with the control).

## 3. Discussion

Despite the widespread use of *Hamamelis virginiana* (witch hazel) in dermatology and cosmetology, there are still relatively few studies focusing on the detailed phytochemistry of this plant. Therefore, the first stage of our research involved a phytochemical analysis of the polyphenolic composition of the leaves and bark. Three different extraction methods were applied to investigate this composition, as the choice of extraction technique often affects the efficiency of isolating biologically active compounds from plant materials. Consequently, the qualitative and quantitative profiles obtained using different extraction approaches may vary [22]. Both the extraction technique and the associated parameters directly influence the yield and quality of the obtained extract. Mild conditions often result in poor extraction efficiency, whereas overly harsh conditions (such as high temperatures or prolonged extraction times) can lead to the degradation of phenolic compounds [23,24,25]. Ultrasound-assisted extraction (UAE), microwave-assisted extraction (MAE), and accelerated solvent extraction (ASE) were used in our study because they offer several advantages over conventional techniques, including shorter extraction times, reduced solvent consumption, and often improved extraction efficiency [26]. The choice of extraction solvents was based on previous reports, which indicate that solvents with high polarity, such as aqueous ethanol or methanol, are typically used to achieve high yields of phenolic compounds [27,28,29]. These solvents effectively extract both simple phenolics and more complex tannins, which are considered the main bioactive constituents of *H. virginiana* extracts.

In our study, the results obtained using optimal conditions for different methods were similar for leaf extraction, and the differences ranged only from a few to several percent. In contrast, the differences between the extraction techniques were more pronounced in the case of bark. Among the methods tested, accelerated solvent extraction (ASE) proved to be the most effective for isolating polyphenolic compounds from the bark of *H. virginiana*. These findings are consistent with previous reports demonstrating the superior efficiency of ASE in recovering polyphenols, especially high molecular weight, from lignified plant matrices [30,31]. This efficiency is attributed to the high pressure and temperature conditions, which enhance solvent penetration and compound solubilization [32]. Moreover, ASE minimizes oxidation and degradation due to shorter extraction times and better control over extraction conditions [32].

In the available literature, there is a noticeable lack of data on the flavonoid content expressed per gram of dried plant material, which serves as the pharmacopoeial raw material (*Hamamelidis folium*), listed in the European Pharmacopoeia. Many authors report results either per mL of extract solution, per gram of dry extract, or based on fresh leaf weight [14,16,33]. As a result, direct comparison of our findings with most published data is not possible. Only a study by Wang et al. provides quantitative values for key metabolites such as hamamelitannin, gallic acid, catechin, and gallocatechin expressed relative to raw plant material [34]. Wang et al. reported 0.21% gallic acid and 0.04% hamamelitannin in leaf, while our analysis yielded 0.14% and 0.03%, respectively. In turn, for bark extracts, the values were 0.59% vs. 0.08% (in our study); for gallic acid, 4.77% vs. 6.3%; for hamamelitannin; and for catechin, 0.39% vs. 0.33%. These differences result from the use of different extraction methods, solvents, and plant material of different origin. It should be highlighted that our paper is the first work providing detailed quantitative data on a wide range of *Hamamelis* metabolites. However, it should be noted that the results for high-molecular-weight tannins are semi-quantitative due to the lack of appropriate standards.

Without a doubt, the abundance of polyphenolic compounds contributes to the biological activity of the extracts observed in this study, as numerous scientific reports indicate that this group of secondary metabolites exhibits potent antioxidant and anti-inflammatory properties [35,36]. These effects are highly valuable in cosmetic formulations, as they play a crucial role in mitigating oxidative stress, which is one of the primary drivers of skin aging and aging-associated inflammation, known as “inflammaging” [37]. The antioxidant activity of polyphenols may be associated not only with direct mechanisms but also with their modulation of cellular enzymes, including superoxide dismutase (SOD), catalase (CAT), and glutathione peroxidase (GPx) [38,39]. These enzymes play complementary and essential roles in the overall antioxidant defense system, protecting cellular components such as lipids, proteins, and DNA against reactive hydroxyl radicals and maintaining cellular redox homeostasis. SOD serves as the first line of defense against reactive oxygen species by converting superoxide anions into hydrogen peroxide and molecular oxygen. This prevents the formation of more reactive species, such as hydroxyl radicals, which can be generated through the Fenton reaction. The hydrogen peroxide produced by SOD is subsequently neutralized by CAT and GPx. CAT decomposes H_2_O_2_ into water and oxygen, acting particularly efficiently at high H_2_O_2_ concentrations. GPx, in turn, reduces both H_2_O_2_ and lipid hydroperoxides using glutathione as a cofactor. It is particularly involved in protecting membrane lipids from peroxidation and contributes to the regulation of redox signaling [40,41].

Our study demonstrated that both bark and leaf extracts showed strong ROS-scavenging effects and significantly reduced ROS level under oxidative stress both in fibroblasts and keratinocytes. These effects were accompanied by a pronounced influence on SOD and by enhancing SOD activity; the extracts may strengthen the first line of cellular defense against excess ROS, thereby preventing oxidative damage to essential cellular macromolecules such as lipids, proteins, and DNA [42,43]. In contrast, the effect on glutathione peroxidase (GPx) was only slight, and no impact on catalase (CAT) activity was observed. A similar pattern was reported by Piazza et al., who found that a glycolic extract from the twigs and bark of *H. virginiana* significantly reduced ROS levels in HaCaT cells without affecting catalase activity [14]. ROS-scavenging activity of witch hazel extract (no data on type of extract) was also observed by Liu et al. in an ex vivo skin model exposed to UVA-induced oxidative stress [15]. Furthermore, the antioxidant potential of different plant extracts was verified using chemical tests like DPPH, ABTS, and ORAC [14,44]. It is worth mentioning that the strong intrinsic antioxidant activity of the extracts observed in our study, and the resulting lowering of the cellular redox state beyond the physiological baseline, may also have adverse consequences. Over-suppression of ROS has been linked to impaired host defense mechanisms and reduced activation of protective stress pathways. Moreover, an excessively reduced intracellular environment may contribute to reductive stress, mitochondrial dysfunction, and altered metabolic homeostasis [45,46].

It should also be pointed out that, at a concentration of 250 µg/mL, the extracts exhibited measurable cytotoxicity in both HaCaT and HDF cells. This limitation may affect the interpretation of the antioxidant data obtained at this dose. The strong reduction in intracellular ROS could partly result from decreased metabolic activity or cell death rather than solely from the intrinsic antioxidant capacity of the extracts. Consequently, the results at 250 µg/mL should be regarded with caution, while the effects observed at lower, non-toxic concentrations provide more reliable evidence of antioxidant activity.

The results of this study also demonstrate that *H. virginiana* leaf and bark extracts exert significant anti-inflammatory effects in LPS-stimulated human dermal fibroblasts (HDFs). This was evident from the decreased levels of key pro-inflammatory cytokines, IL-6, IL-1β, and TNF-α, following treatment with both tested concentrations (50 and 125 µg/mL), with the most pronounced effects observed at the lower concentration of 50 µg/mL. These findings are consistent with those of previous studies highlighting the anti-inflammatory potential of *H. virginiana*. For instance, Piazza et al. demonstrated that a standardized bark glycolic extract suppressed the release of pro-inflammatory and allergic mediators (e.g., IL-6, IL-17C, TSLP, CCL26) in cytokine-stimulated human keratinocytes, partly through inhibition of NF-κB signaling and restoration of skin differentiation markers [47]. In a separate study, the same group reported that the extract reduced IL-6 and IL-8 levels in *Cutibacterium acnes*-infected keratinocytes [14]. Similarly, Amêndola et al. showed that *H. virginiana* leaf extracts reduced IL-1β, TNF-α, and nitric oxide production in LPS-stimulated murine macrophages [48]. Our observation that lower concentrations of the extracts resulted in stronger anti-inflammatory effects is in line with previous reports suggesting that excessive doses of polyphenol-rich plant extracts can paradoxically induce oxidative stress and stimulate inflammatory signaling pathways [49,50]. This biphasic response is a well-recognized phenomenon known as hormesis.

Another important aspect of witch hazel activity on the skin is its impact on matrix metalloproteinases (MMPs), such as collagenase, hyaluronidase, and elastase. These enzymes play a key role in the degradation of extracellular matrix components, including collagen and elastin, which are essential for maintaining skin structure and elasticity. Their excessive activity contributes to skin aging, wrinkle formation, and loss of firmness. Therefore, inhibition of these enzymes is associated with protective and anti-aging effects on the skin [51,52]. Our study demonstrated that the ethanolic extract of *Hamamelis* bark exhibits potent inhibitory activity against elastase and, at higher concentrations, also against collagenase, confirming its potential usefulness in cosmetology. Until now, only one study has investigated this type of activity in the *Hamamelis* plant. Thring et al. reported that a water extract from the leaves showed only weak or no anti-collagenase and anti-elastase activity [53], which was also confirmed in our investigation. The inhibitory properties of the bark extract were probably attributed to its higher tannin content, as literature data report such activity for tannin-class compounds, such as EGCG [53,54].

Interestingly, while both leaf and bark extracts of *H. virginiana* demonstrated comparable antioxidant and anti-inflammatory activities, a moderate cytotoxic effect was observed at higher concentrations of the bark extract. This may be linked to its elevated tannin levels or, conversely, to the presence of flavonoids in the leaf extract, which are known for their cytoprotective effects. Such protective properties of flavonoids have been previously reported [55,56].

Overall, the findings highlight the promising role of *H. virginiana* leaf and bark extracts as active components in skincare formulations, offering protection to skin cells from oxidative damage and inflammation, with the bark extract additionally helping to prevent the breakdown of the extracellular matrix.

## 4. Materials and Methods

### 4.1. Extract Preparation and Phytochemical Characterization

Leaves and bark of *Hamamelis virginiana* (witch hazel) were purchased from commercial sources in three separate batches obtained from different suppliers. The individual batches were combined to ensure sample uniformity and subsequently ground into a fine powder using a laboratory mill (IKA M20, IKA-Werke GmbH & Co. KG, Staufen, Germany). Extraction was performed separately for leaves and bark using three different extraction methods: heat reflux extraction (HRE) employing a heating block (Julabo MB-5, Julabo GmbH, Seelbach, Germany), ultrasound-assisted extraction (UAE) using an ultrasonic bath (Bandelin Sonorex Digitec, Bandelin Electronic GmbH & Co. KG, Berlin, Germany), and accelerated solvent extraction (ASE) with a Dionex ASE 350 extractor (Thermo Fisher Scientific Inc., Sunnyvale, CA, USA). For each method, four solvent systems were applied: 100% methanol, methanol/water (8:2, *v*/*v*), methanol/water (6:4, *v*/*v*), and methanol/water (4:6, *v*/*v*). Extraction temperature was set at 80 °C. The duration of each extraction step was 30 min for HRE and 15 min for UAE. For ASE, the extraction procedure consisted of a static extraction time of 5 and 10 min. Extraction completeness was confirmed analytically by monitoring the presence of polyphenols as the target compounds using ultra-high-performance liquid chromatography (UPLC). The resulting extracts were combined. For biological studies, the plant material was extracted using a two-step ASE procedure with ethanol, followed by 80% ethanol as the extraction solvents. The extracts were lyophilized using a Christ Alpha 2–4 LDplus freeze dryer (Martin Christ Gefriertrocknungsanlagen GmbH, Osterode am Harz, Germany) and stored at −20 °C until further use.

### 4.2. Biological Assays

#### 4.2.1. Cytotoxicity Analysis

##### Cell Culture

The cytotoxic potential of the tested extracts and ferments was evaluated using two human skin-derived cell lines: human dermal fibroblasts (HDFs) and immortalized human keratinocytes (HaCaTs). Cells were maintained in Dulbecco’s Modified Eagle Medium (DMEM) supplemented with 10% fetal bovine serum (FBS) and 1% penicillin–streptomycin and incubated under standard conditions (37 °C, 5% CO_2_). Once the cultures reached approximately 70–80% confluency, they were detached using 0.25% trypsin-EDTA solution and seeded into 96-well microplates at a density suitable for viability assays. After allowing cells to adhere overnight, they were exposed to various concentrations of the test substances for the subsequent viability assessment.

##### Alamar Blue

To assess mitochondrial metabolic activity, the Alamar Blue assay was employed. A working solution of 60 µM resazurin in culture medium was freshly prepared and dispensed into each well containing the treated cells. Parallel wells with untreated cells cultured solely in DMEM were included as negative controls. Following a 2 h incubation period at 37 °C, fluorescence intensity was measured using a microplate reader (Thermo Fisher Scientific, Waltham, MA, USA) with excitation/emission settings at 570 nm. The assay was carried out in three biologically independent replicates, and the results were normalized to the untreated control, expressed as a percentage to facilitate comparison across experimental conditions [57].

##### Neutral Red

The Neutral Red assay was conducted to determine the functional integrity of lysosomal membranes in viable cells. After exposure, cells were incubated with a neutral red staining solution prepared in DMEM. This dye selectively accumulates in lysosomes of living cells, providing a marker of cellular viability. After a 2 h incubation, the wells were gently rinsed with pre-warmed sterile phosphate-buffered saline (PBS) to remove excess dye, followed by the addition of a destaining solution to extract the accumulated dye from the cells. Absorbance was subsequently recorded at 540 nm using a microplate reader (Thermo Fisher Scientific, Waltham, MA, USA). All experiments were performed in triplicate, and the absorbance values were expressed as a percentage of the control group.

#### 4.2.2. Determination of Intracellular ROS Levels in Human Skin Cells

The antioxidative potential of *H. virginiana* extracts was investigated by quantifying the intracellular levels of reactive oxygen species (ROS) in human keratinocytes (HaCaTs) and dermal fibroblasts (HDFs). The analysis was carried out using the fluorescent probe 2′,7′-dichlorodihydrofluorescein diacetate (H_2_DCFDA; Merck KGaA, Darmstadt, Germany). HaCaT and HDF cells were seeded separately in black 96-well microplates (Merck KGaA, Darmstadt, Germany) at a density of 1 × 10^4^ cells per well and incubated in Dulbecco’s Modified Eagle Medium (DMEM; Merck KGaA, Darmstadt, Germany) supplemented with 10% fetal bovine serum (FBS; Genos, Łódź, Poland) for 24 h at 37 °C in a humidified atmosphere containing 5% CO_2_. After this initial incubation, the culture medium was removed and replaced with fresh DMEM containing various concentrations (50, 125 and 250 μg/mL) of *H. virginiana* leaf or bark extracts. The cells were exposed to the extracts for an additional 24 h under standard culture conditions. Following incubation with the extracts, the medium was aspirated and replaced with serum-free DMEM containing 10 μM H_2_DCFDA. To induce oxidative stress, hydrogen peroxide (H_2_O_2_; Merck KGaA, Darmstadt, Germany) was simultaneously added to each well to achieve a final concentration of 500 μM. Negative control wells contained cells not treated with either H_2_O_2_ or extracts, while positive control wells were exposed to H_2_O_2_ alone. After 60 min of incubation with the fluorescent probe at 37 °C, intracellular fluorescence intensity was measured using a multi-mode microplate reader (FilterMax F5, Thermo Fisher Scientific, Waltham, MA, USA) at an excitation wavelength of 485 nm and emission at 530 nm. All measurements were performed in triplicate within three independent experiments.

#### 4.2.3. Antioxidant Enzymes Activity

To induce oxidative stress, cells were treated with hydrogen peroxide (H_2_O_2_) at a final concentration of 500 μM, while simultaneously being exposed to the test extracts. Following a 6 h incubation at 37 °C under standard culture conditions (5% CO_2_, humidified atmosphere), malondialdehyde (MDA) levels and the enzymatic activities of superoxide dismutase (SOD), glutathione peroxidase (GPx), and catalase (CAT) were determined. All measurements were performed using commercial assay kits (Abcam, Berlin, Germany), in accordance with the manufacturer’s protocol.

#### 4.2.4. Assessment of Anti-Inflammatory Activity

To assess the anti-inflammatory potential of leaf and bark extracts derived from *H. virginiana*, levels of the pro-inflammatory cytokines IL-6 IL-1β, and TNF-α were measured in human dermal fibroblasts (HDFs) following stimulation with lipopolysaccharide (LPS, 10 µg/mL) from *E. coli* O111:B4 for a duration of 24 h. Simultaneously, the cells were treated with the test compounds at a concentration of 50 and 125 µg/mL. After incubation, the culture medium (DMEM) was removed, and cells were washed with phosphate-buffered saline (PBS) and lysed using RIPA buffer. The resulting lysates were subjected to ELISA analysis (Elabscience, Biotechnology Inc., Houston, TX, USA) following the manufacturer’s protocol. Absorbance readings were obtained at 450 nm using a FilterMax F5 microplate reader (Thermo Fisher Scientific, Waltham, MA, USA). Untreated cells served as the negative control, while those exposed only to LPS represented the positive control.

#### 4.2.5. Assessment of Extracellular Matrix (ECM) Degrading Enzymes Activity

In order to determine the ability of *H. virginiana* L. leaf and bark extract, the enzymatic activity of collagenase neutrophil elastase and hyaluronidase was examined using spectrophotometric analyses performed with the human COL2 α 1 ELISA kit, human NE/ELA2 ELISA kit, and human HAase ELISA kit (Elabscience Biotechnology Inc., Houston, TX, USA) according to the manufacturer’s instructions. Human fibroblasts were cultured, treated with the extract and ferments (at concentrations of 50 and 125 µg/mL), lysed, and subjected to ELISA protocols. Absorbance measurements at a wavelength of 450 nm were performed using a microplate reader (Thermo Fisher Scientific, Waltham, MA, USA). The results were expressed as fold compared with the control (HDF cells not treated with test compounds). To confirm the inhibitory effects, reference inhibitors were included as positive controls. For elastase, succinyl–alanyl–alanyl–prolyl–valyl chloromethyl ketone (SPCK; Adooq Bioscience, Irvine, CA, USA) was applied at a final concentration of 30 µM. Collagenase activity was assessed in the presence of 1,10-phenanthroline (Abcam, Cambridge, UK) at 300 µM. In the case of hyaluronidase, tannic acid (Merck KGaA, Darmstadt, Germany) at a concentration of 300 µM was used as the control inhibitor.

## 5. Conclusions

In our work, the detailed phytochemical profile bark and leaf from *H. virginiana* plants were established using UPLC-DAD-MS profiling. Furthermore, this study demonstrated that ethanolic extracts from the leaves and bark of the plant possess strong antioxidant and anti-inflammatory properties. Both extracts effectively reduced ROS levels in fibroblasts and keratinocytes and enhanced SOD activity, suggesting protection against oxidative stress. They also significantly decreased key pro-inflammatory cytokines (IL-6, IL-1β, TNF-α) in stimulated fibroblasts, with more pronounced effects observed at lower concentrations, indicating a possible hormetic response. Additionally, the bark extract showed potent inhibitory activity against collagenase and elastase. Taken together, these findings support the potential use of *H. virginiana* extracts in cosmetology as protective and anti-aging agents.

However, further studies are needed to fully explore the potential of *H. virginiana*. More detailed mechanistic investigations could help clarify the molecular pathways through which *H. virginiana* extracts exert their protective effects. In vivo studies and clinical trials will also be crucial to validate the observed bioactivities under physiological conditions and to establish safety profiles for long-term topical use. Finally, optimized extraction strategies should be developed to ensure eco-friendly and economically viable production of extracts rich in bioactive ingredients.

From an application standpoint, the extracts show strong potential as additives in cosmeceutical formulations aimed at skin protection, anti-aging, and soothing of sensitive or inflamed skin. Their dual antioxidant and anti-inflammatory activities make them particularly attractive for incorporation into creams, serums, or gels designed to counteract environmental stressors such as UV radiation and pollution, as well as into formulations targeting skin firmness and elasticity. Beyond cosmetics, *H. virginiana* extracts may also serve as adjunct therapies for managing inflammatory skin disorders such as eczema, psoriasis, or acne.

## Figures and Tables

**Table 1:** Retention times and mass spectrometry data for the components identified in bark (B) and leaf (L) extracts of *H. virginiana*.

RT (min.)	Mass Data (*m*/*z*-H)	Formula	Δ ppm	Component	B	L	Ref.
3.25	331.06739	C_13_H_16_O_10_	0.96	Galloyl-hexose	+	+	[16]
3.87	331.06758	C_13_H_16_O_10_	1.53	Galloyl-hexose	+	+	[16]
4.68	169.01498	C_7_H_6_O_5_	4.31	Gallic acid	+	+	[16], str
4.61	331.06791	C_13_H_16_O_10_	2.53	Galloyl-hexose	+	+	[16]
5.83	331.06761	C_13_H_16_O_10_	1.63	Galloyl-hexose	+	+	[16]
5.93	343.06634	C_14_H_16_O_10_	−2.12	Galloylquinic acid	+	+	tv
6.93	343.06621	C_14_H_16_O_10_	−2.50	Galloylquinic acid	+	+	tv
7.13	331.06771	C_13_H_16_O_10_	1.93	Galloyl-hexose	+	+	[16]
8.48	243.05069	C_10_H_12_O_7_	−1.38	Galloylglycerol	+	+	tv
8.68	153.01921	C_7_H_6_O_4_	−0.79	Protocatechuic acid	+	+	str
8.73	483.07857	C_20_H_20_O_14_	1.12	Digalloyl hexose	+	+	[16]
9.03	315.07335	C_13_H_16_O_9_	3.78	Hydroxybenzoic acid hexoside	+	-	tv
9.37	305.06764	C_15_H_14_O_7_	3.15	Gallocatechin	+	+	[16]
10.77	483.07875	C_20_H_20_O_14_	1.49	Digalloyl hexose	+	+	[16]
11.16	353.08892	C_16_H_18_O_9_	3.15	Neochlorogenic acid	+	+	[17], str
11.55	483.07894	C_20_H_20_O_14_	1.88	Digalloyl hexose	+	+	[16]
13.08	483.07899	C_20_H_20_O_14_	1.99	Digalloyl hexose	+	-	[16]
13.50	183.02983	C_8_H_8_O_5_	−0.36	Methyl gallate	+	+	tv
13.53	337.09377	C_16_H_18_O_8_	2.60	3-p-Coumaroylquinic acid	-	+	[17]
13.79	321.02382	C_14_H_10_O_9_	−4.30	Digallic acid	-	+	[16]
14.24	337.09359	C_16_H_18_O_8_	2.07	3-p-Coumaroylquinic acid	-	+	[17]
14.50	577.13258	C_30_H_26_O_12_	−4.45	Procyanidin B2	+	-	[16]
14.87	495.07649	C_21_H_20_O_14_	−3.10	di-O-galloylquinic acid	+	-	tv
15.53	289.07221	C_15_H_14_O_6_	1.55	Catechin	+	+	[16,17], str
15.71	635.08891	C_27_H_24_O_18_	−0.12	Tri-O-galloyl-hexose	+	-	[16]
16.23	483.07905	C_20_H_20_O_14_	2.11	Hamamelitannin	+	+	[16,17], str
16.26	353.08847	C_16_H_18_O_9_	1.88	Chlorogenic acid	-	+	[17], str
17.13	321.02400	C_14_H_10_O_9_	−3.74	Digallic acid	-	+	[16]
17.68	635.08904	C_27_H_24_O_18_	0.08	Tri-O-galloyl-hexose	-	+	[16]
19.49	289.07246	C_15_H_14_O_6_	2.41	Epicatechin	+	+	str
19.76	337.09381	C_16_H_18_O_8_	2.72	4-p-Coumaroylquinic acid	-	+	[17]
20.36	337.09367	C_16_H_18_O_8_	2.30	5-p-Coumaroylquinic acid	-	+	[17]
21.55	335.07880	C_16_H_16_O_8_	4.64	Caffeoylshikimic acid	-	+	[17]
21.66	635.08862	C_27_H_24_O_18_	−0.58	Tri-O-galloyl-hexose	+	+	[16]
22.73	337.09359	C_16_H_18_O_8_	2.07	5-p-Coumaroylquinic acid	-	+	[17]
23.59	483.07869	C_20_H_20_O_14_	1.37	Di-O-galloyl-hexose	+	+	[16]
24.74	635.08924	C_27_H_24_O_18_	0.40	Tri-O-galloyl-hexose	+	+	[16]
24.96	335.04099	C_15_H_12_O_9_	0.40	Galloyl gallic acid methyl ester	+	+	tv
25.41	441.08263	C_22_H_18_O_10_	−0.20	Catechin gallate	+	+	tv
26.03	787.09787	C_34_H_28_O_22_	−2.63	Tetra-O-galloylhexose	-	+	[16]
29.49	615.10137	C_28_H_24_O_16_	3.59	Quercetin-galloyl hexoside	-	+	[17]
29.75	609.14527	C_27_H_30_O_16_	−1.37	Quercetin rhamnoside-hexoside	-	+	tv
30.18	787.09789	C_34_H_28_O_22_	−2.61	Tetra-O-galloylhexose	+	+	[16]
30.53	300.99853	C_14_H_6_O_8_	−1.53	Ellagic acid	+	+	[17], str
30.59	441.08274	C_22_H_18_O_10_	0.04	Epicatechin gallate	+	+	tv
30.89	609.14503	C_27_H_30_O_16_	−1.77	Quercetin rhamnoside-hexoside	-	+	tv
31.80	787.09845	C_34_H_28_O_22_	−1.90	Tetra-O-galloyl-hexose	+	+	[16]
32.09	609.14487	C_27_H_30_O_16_	−2.03	Quercetin 3-O-rutinoside	-	+	[17], str
32.54	463.08837	C_21_H_20_O_12_	0.37	Quercetin 3-O-galactoside	-	+	str
33.49	477.06739	C_21_H_18_O_13_	−0.16	Quercetin-3-O-glucuronide	-	+	str
33.71	463.08891	C_21_H_20_O_12_	1.53	Quercetin 3-O-glucoside	-	+	str
33.41	787.09874	C_34_H_28_O_22_	−1.53	Tetra-O-galloyl-hexose	+	+	[17]
34.34	335.04090	C_15_H_12_O_9_	0.13	galloyl gallic acid methyl ester	-	+	tv
34.72	593.15175	C_27_H_30_O_15_	0.94	Kaempferol rhamnoside-hexoside	-	+	tv
35.05	593.15207	C_27_H_30_O_15_	1.47	Kaempferol rhamnoside-hexoside	-	+	tv
37.28	447.09349	C_21_H_20_O_11_	0.46	Kaempferol 3-O-galactoside	-	+	[17], str
38.11	593.15169	C_27_H_30_O_15_	0.84	Kaempferol 3-O-rutinoside	-	+	[17], str
38.29	599.10502	C_28_H_24_O_15_	1.29	Kaempferol galloyl-hexose	-	+	[17]
39.39	447.09361	C_21_H_20_O_11_	0.73	Kaempferol 3-O-glucoside	-	+	[17], str
39.60	447.09361	C_21_H_20_O_11_	0.73	Quercetin 3-O-rhamnoside	-	+	str
39.26	939.10949	C_41_H_32_O_26_	−1.50	Penta-O-galloyl-hexose	+	+	[17]
44.26	1091.12159	C_48_H_36_O_30_	−0.25	Hexa-O-galloyl-hexose	+	+	[16,17]
45.06	1091.12191	C_48_H_36_O_30_	0.04	Hexa-O-galloyl-hexose	+	+	[16,17]
45.91	1091.12241	C_48_H_36_O_30_	0.50	Hexa-O-galloyl-hexose	+	+	[16,17]
48.98	1243.13238	C_55_H_40_O_34_	−0.36	Hepta-O-galloyl-hexose	-	+	[16,17]
51.39	301.03585	C_15_H_10_O_7_	1.57	Quercetin	-	+	[16,17], str
52.62	1395.14456	C_62_H_44_O_38_	0.56	Octa-O-galloyl-hexose	-	+	[16,17]
55.34	593.13084	C_30_H_26_O_13_	1.31	Tiliroside	-	+	str
59.13	1547.15599	C_69_H_48_O_42_	0.81	Nona-O-galloyl-hexose	-	+	[16,17]
59.61	285.04152	C_15_H_10_O_6_	3.70	Kaempferol	-	+	[16,17], str

Str—confirmed by standard; tv—tentatively identified; “+”—detected; “-”—not detected.

**Table 2:** The results of quantification of the main metabolites found in *H. virginiana* leaf extract, expressed as µg per gram of dried plant material (±SD).

Compound	HRE	UAE	ASE
Tannins			
Galloyl hexoses **	121.8 ± 27.3 ^a^	115.2 ± 10.6 ^a^	129.1 ± 11.7 ^a^
Galloylquinic acids *	449.1 ± 30.8 ^a^	271.0 ± 19.2 ^c^	366.2 ± 16.3 ^b^
Hamamelitannin	298.9 ± 17.0 ^a^	240.2 ± 9.8 ^b^	275.1 ± 13.2 ^a^
Digallic acid **	258.7 ± 17.0 ^b^	228.4 ± 14.8 ^b^	434.8 ± 23.8 ^a^
Digalloyl hexoses **	83.3 ± 4.8 ^a^	57.2 ± 3.7 ^c^	71.2 ± 5.2 ^b^
Tri-O-galloylhexoses **	312.8 ± 17.3 ^a^	278.3 ± 18.7 ^b^	177.6 ± 7.8 ^c^
Galloyl gallic acid methyl esters	401.9 ± 28.8 ^a^	255.4 ± 13.9 ^b^	374.2 ± 29.6 ^a^
Tetra-O-galloylhexoses **	1282.7 ± 78.4 ^a^	1061.9 ± 61.0 ^b^	1030.9 ± 60.6 ^b^
Penta-O-galloylhexoses **	5596.4 ± 351.0 ^a^	4294.9 ± 291.6 ^b^	5321.2 ± 420.7 ^a^
Hexa-O-galloylhexoses **	7347.3 ± 565.9 ^a^	5178.7 ± 219.6 ^b^	6983.7 ± 448.3 ^a^
Hepta-O-galloylhexoses **	5990.9 ± 256.7 ^a^	4405.0 ± 293.7 ^b^	6301.9 ± 508.0 ^a^
Octa-O-galloylhexoses **	6351.8 ± 276.2 ^a^	5562.5 ± 371.7 ^b^	6685.2 ± 361.0 ^a^
Nona-O-galloylhexoses **	905.1 ± 36.9 ^a^	282.0 ± 13.7 ^c^	399.3 ± 20.5 ^b^
Total (mg/g)	29.40 ± 2.10 ^a^	22.23 ± 2.03 ^b^	28.55 ± 2.24 ^a^
Phenolic acids			
Gallic acid	1219.4 ± 75.5 ^b^	1061.3 ± 53.6 ^c^	1375.4 ± 63.7 ^a^
Protocatechuic acid	286.8 ± 18.4 ^a^	244.8 ± 14.3 ^b^	263.9 ± 17.5 ^a,b^
Chlorogenic acids	344.4 ± 21.3 ^a^	340.3 ± 21.4 ^a^	360.6 ± 16.4 ^a^
Methyl gallate *	1590.6 ± 120.4 ^b^	1115.5 ± 72.1 ^c^	1969.1 ± 86.4 ^a^
p-Coumaroylquinic acids ***	489.7 ± 38.5 ^b^	484.1 ± 37.6 ^b^	563.0 ± 41.4 ^a^
Ellagic acid	3137.8 ± 230.0 ^b^	2659.8 ± 120.1 ^c^	4796.1 ± 214.9 ^a^
Total (mg/g)	7.07 ± 0.54 ^b^	5.91 ± 0.39 ^c^	9.33 ± 0.61 ^a^
Flavonols			
Quercetin 3-O-rutinoside	167.8 ± 11.9 ^b^	165.3 ± 8.7 ^b^	199.3 ± 10.3 ^a^
Quercetin 3-O-galactoside	109.2 ± 8.2 ^b^	106.0 ± 5.8 ^b^	133.2 ± 6.0 ^a^
Quercetin 3-O-glucuronide	224.3 ± 17.8 ^b^	206.6 ± 13.0 ^b^	292.5 ± 15.4 ^a^
Quercetin 3-O-glucoside	186.7 ± 13.4 ^b^	180.4 ± 10.4 ^b^	227.2 ± 12.9 ^a^
Quercetin 3-O-rhamnoside	123.9 ± 9.6 ^b^	123.0 ± 6.2 ^b^	241.2 ± 10.6 ^a^
Kaempferol 3-O-galactoside	300.3 ± 17.6 ^b^	276.9 ± 22.0 ^b^	359.0 ± 15.3 ^a^
Kaempferol rhamnoside-hexosides	309.6 ± 10.1 ^a^	207.6 ± 6.3 ^b^	340.1 ± 11.3 ^a^
Kaempferol 3-O-rutinoside	204.3 ± 14.6 ^b^	184.4 ± 8.1 ^b^	243.5 ± 16.5 ^a^
Kaempferol 3-O-glucoside	449.5 ± 20.1 ^b^	468.2 ± 22.6 ^b^	534.0 ± 33.5 ^a^
Tiliroside	97.5 ± 4.4 ^b^	87.1 ± 4.0 ^c^	131.2 ± 6.6 ^a^
Kaempferol	73.8 ± 3.4 ^b^	58.4 ± 3.9 ^c^	96.0 ± 5.9 ^a^
Total (mg/g)	2.25 ± 0.19 ^b^	2.06 ± 0.16 ^b^	2.80 ± 0.18 ^a^

* calculation was based on gallic acid calibration curve; ** calculation was based on hamamelitannin calibration curve; *** calculation was based on p-coumaric acid calibration curve; HRE—heat reflux extraction; UAE—ultrasonic assisted extraction; ASE—accelerated solvent extraction; different lowercased letters indicate that the differences were statistically significant.

**Table 3:** The results of quantification of the main metabolites found in *H. virginiana* bark extract, expressed as µg per gram of dried plant material (±SD).

Compound	HRE	UAE	ASE
Tannins			
Galloyl hexoses **	6465.8 ± 366.5 ^a^	6537.2 ± 268.6 ^a^	6602.4 ± 308.9 ^a^
Galloylquinic acids *	3442.4 ± 137.7 ^a,b^	3291.8 ± 186.6 ^b^	3667.1 ± 101.1 ^a^
Di-O-galloylhexoses **	893.7 ± 42.4 ^a,b^	831.3 ± 59.9 ^b^	940.8 ± 43.7 ^a^
Tri-O-galloylhexoses **	3701.1 ± 206.8 ^c^	3835.6 ± 201.5 ^b^	4516.0 ± 204.7 ^a^
Tetra-O-galloylhexoses **	722.1 ± 44.4 ^b^	610.4 ± 41.3 ^c^	1299.1 ± 95.3 ^a^
Penta-O-galloylhexoses **	1108.1 ± 62.9 ^b^	1140.6 ± 85.6 ^b^	2261.8 ± 72.1 ^a^
Hexa-O-galloylhexoses **	199.5 ± 13.4 ^b^	131.2 ± 9.9 ^c^	367.7 ± 25.7 ^a^
di-O-galloylquinic acid **	348.3 ± 26.2 ^b^	330.7 ± 13.7 ^b^	552.7 ± 38.7 ^a^
Hamamelitannin	48,230.7 ± 2107.4 ^b^	49,139.8 ± 2355.0 ^b^	62,749.6 ± 2461.5 ^a^
Total (mg/g)	65.11 ± 4.47 ^b^	65.85 ± 5.05 ^b^	82.96 ± 6.85 ^a^
Phenolic acids			
Gallic acid	619.7 ± 42.6 ^b^	590.8 ± 39.5 ^b^	787.0 ± 49.1 ^a^
Protocatechuic acid	106.3 ± 5.5 ^a,b^	110.6 ± 6.9 ^a^	97.2 ± 3.5 ^b^
Methyl gallate *	456.4 ± 24.6 ^b^	334.2 ± 26.3 ^c^	543.4 ± 24.6 ^a^
Ellagic acid	547.9 ± 26.4 ^c^	622.4 ± 27.0 ^b^	1071.2 ± 75.2 ^a^
Total (mg/g)	0.17 ± 0.02 ^b^	0.17 ± 0.02 ^b^	0.25 ± 0.02 ^a^
Flavan-3-ols			
Catechin	2910.0 ± 133.5 ^b^	2755.7 ± 125.7 ^c^	3345.4 ± 152.0 ^a^

* calculation was based on gallic acid calibration curve; ** calculation was based on hamamelitannin calibration curve; HRE—heat reflux extraction; UAE—ultrasonic assisted extraction; ASE—accelerated solvent extraction; different lowercased letters indicate that the differences were statistically significant.

## Data Availability

The data presented in this study are available on request from the corresponding author.

## Supplementary Materials

The following supporting information can be downloaded at https://www.mdpi.com/article/10.3390/molecules30173572/s1: Table S1: The results of the quantitative analysis (mg/g ± standard deviation) of the main components in the dried bark and leaf extracts of *H. virginiana*. Figure S1: Representative UV–VIS and MS spectra of specific components characteristic of particular classes of metabolites. Figure S2: Extracted ion chromatograms in the mass range specific for flavonoid compounds. Figure S3: Base ion chromatograms of *H. virginiana* leaf extracts obtained using HRE—heat reflux extraction (red), UAE—ultrasonic assisted extraction (blue), and ASE—accelerated solvent extraction (green). Figure S4: Base ion chromatograms of *H. virginiana* bark extracts obtained using HRE—heat reflux extraction (red), UAE—ultrasonic assisted extraction (blue), and ASE—accelerated solvent extraction (green).
