# Supplementary material for: Comparative Analysis of Polyphenol-Rich Extracts from Hamamelis virginiana Leaves and Bark: ROS Scavenging and Anti-Inflammatory Effects on Skin Cells
Source: Molecules. 2025 Aug 31;30(17):3572. doi: 10.3390/molecules30173572 (PMC12430692; doi:10.3390/molecules30173572)
Supplement: Supplementary file 1 [file molecules-30-03572-s001.zip › molecules-3833653-supplementary.pdf]

molecules

MDPI

Supplementary Materials

Comparative Analysis of Polyphenol-Rich Extracts from *Hamamelis virginiana* Leaves and Bark: ROS Scavenging and Anti-inflammatory Effects on Skin Cells

Magdalena Wójciak [1]*, Wiktoria Pacuła [1], Katarzyna Tyszczuk-Rotko [2], Aleksandra Ziemlewska [3], Martyna Zagórska-Dziok [3], Zofia Nizioł-Łukaszewska [3], Rafał Patryn [4], Anna Pacian [5], and Ireneusz Sowa [1]*

Table S1. The results of the quantitative analysis (mg/g ± standard deviation) of the main components in the dried bark and leaf extracts of *H. virginiana*.

Component	Bark	Leaf
Galloyl-hexoses	43.78 ± 2.82	0.84 ± 0.02
Gallic acid	4.52 ± 0.32	8.96 ± 0.41
Galloylquinic acid	16.79 ± 1.21	2.07 ± 0.12
Protocatechuic acid	0.47 ± 0.03	1.68 ± 0.76
Digalloyl hexoses	4.92 ± 0.28	0.44 ± 0.02
Chlorogenic acids	det	2.07 ± 0.14
Methyl gallate	2.98 ± 1.73	12.32 ± 1.01
Digallic acids	nd	2.44 ± 0.16
p-coumaroylquinic acids	nd	3.16 ± 0.18
di-O-galloylquinic acid	3.03 ± 0.24	nd
Catechin/Epicatechin	24.68 ± 1.72	0.26 ± 0.02
Tri-O-galloylglucoses	21.53 ± 1.45	1.28 ± 0.09
Hamamelitanin	243.58 ± 18.12	2.29 ± 0.11
galloyl gallic acid methyl esters	det	3.43 ± 0.18
Tetra-O-galloylhexoses	7.21 ± 0.53	5.06 ± 0.36
Penta-O-galloylglucose	11.39 ± 0.87	37.2 ± 2.81
Hexa-O-galloylhexose	1.02 ± 0.08	52.1 ± 3.25
Hepta-O-galloylhexose	det	31.5 ± 2.45
Octa-O-galloylhexose	nd	24.74 ± 1.87
Ellagic acid	4.05 ± 0.31	21.52 ± 1.23
Quercetin 3-O-rutinoside	nd	1.39 ± 0.11
Quercetin 3-O-galactoside	nd	0.73 ± 0.06
Quercetin-3-O-glucuronide	nd	1.63 ± 0.09
Quercetin 3-O-glucoside	nd	1.81 ± 0.12
Quercetin 3-O-rhamnoside	nd	1.49 ± 0.10
Kaempferol 3-O-galactoside	nd	2.07 ± 0.17
Kaempferol 3-O-rutinoside	nd	1.47 ± 0.12
Kaempferol 3-O-glucoside	nd	4.42 ± 0.26
Kaempferol	nd	0.51 ± 0.05

nd - not detected

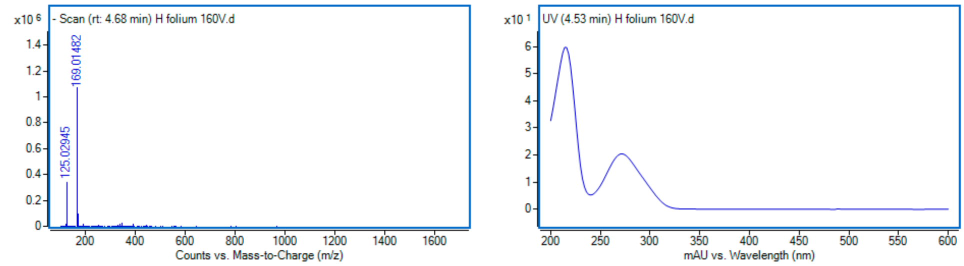

Gallic acid

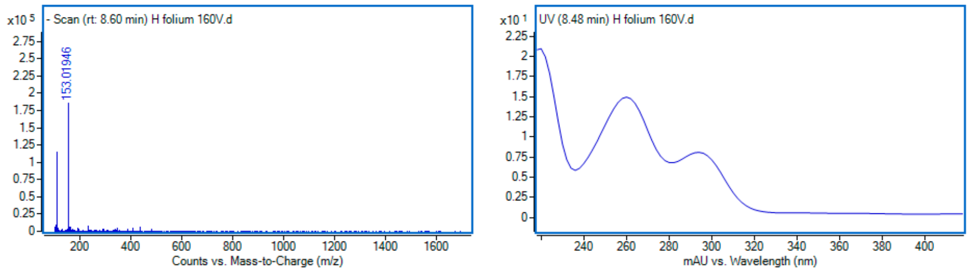

Protocatechuic acid

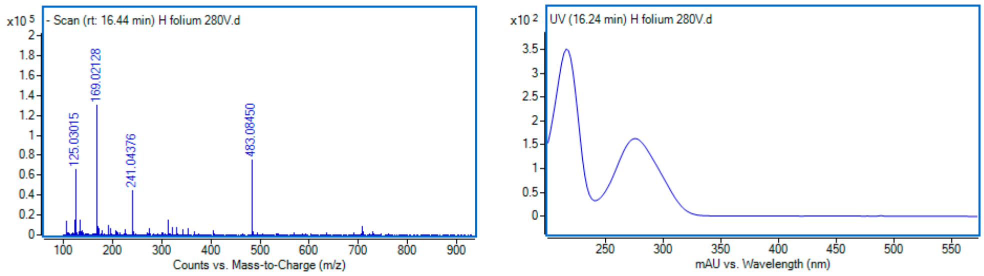

Hamamelitanin

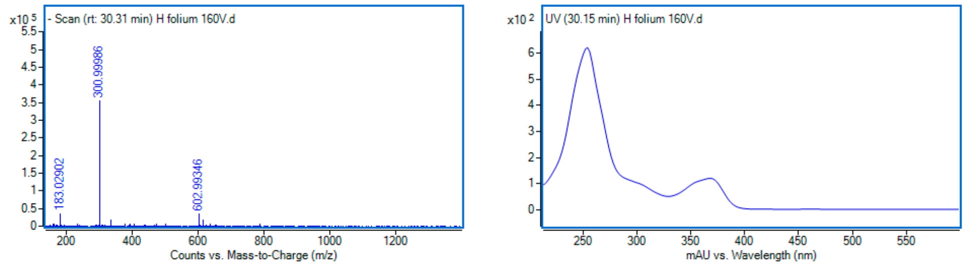

Ellagic acid

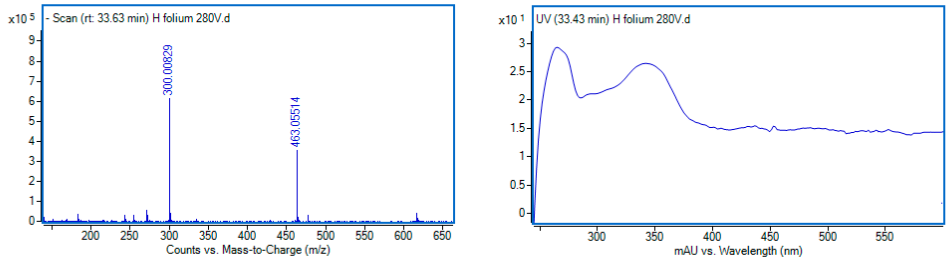

Quercetin 3-O-glucoside

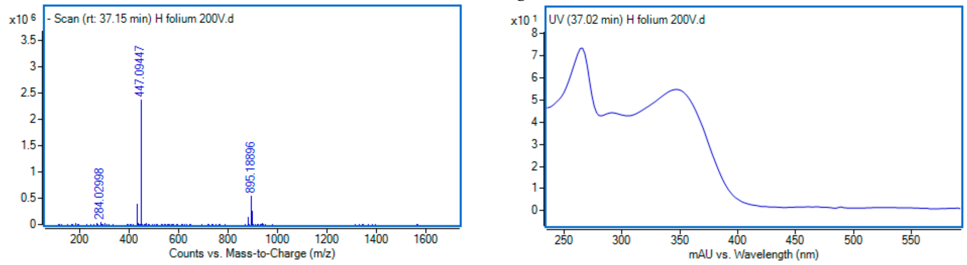

Kaempferol 3-O-galactoside

Figure S1. Representative UV-Vis and MS spectra of specific components characteristic of particular classes of metabolites.

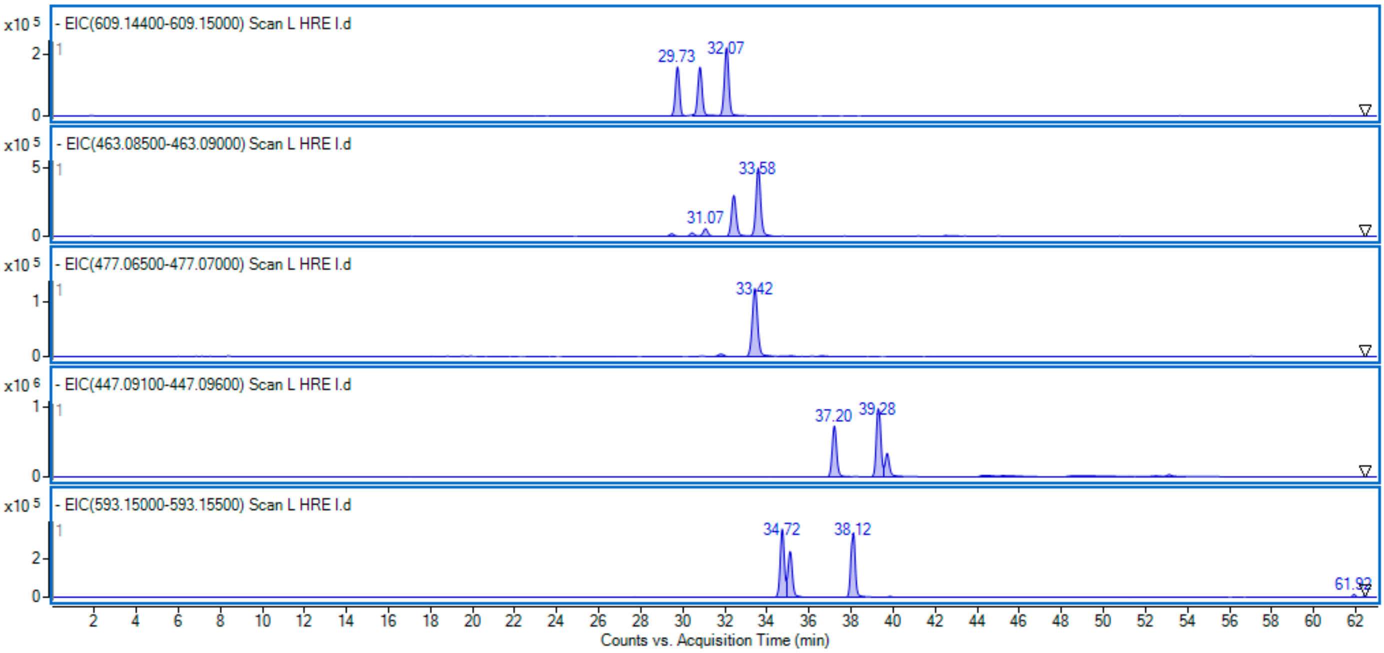

Figure S2. Extracted ion chromatograms in the mass range specific for flavonoid compounds.

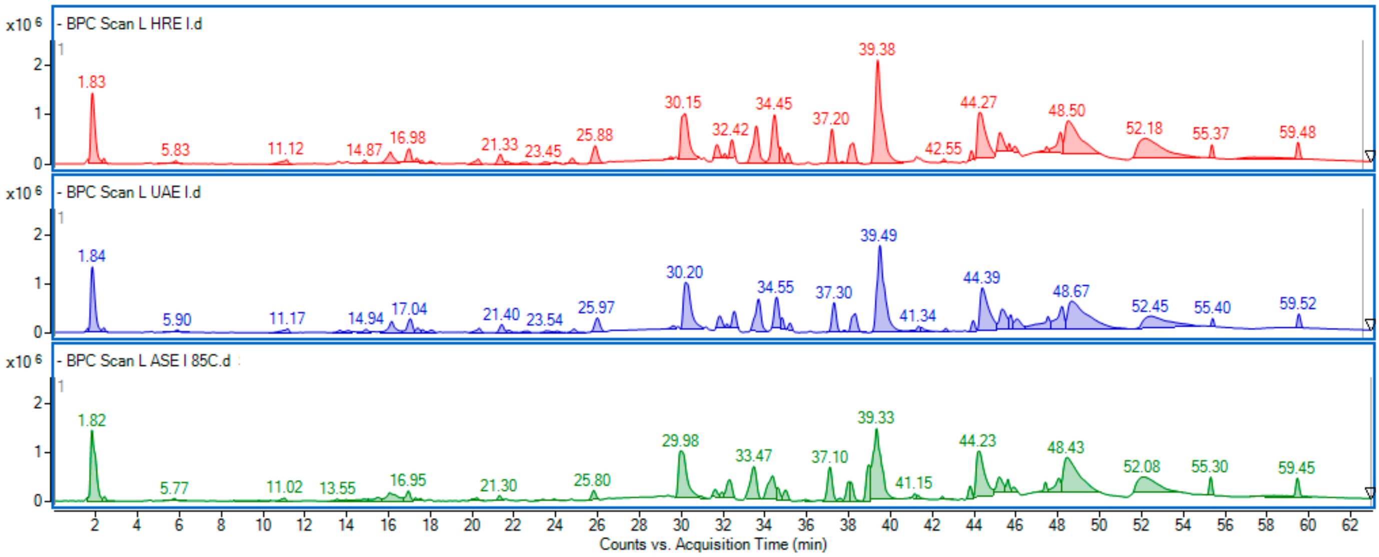

Figure S3. Base ion chromatograms of *H. virginiana* leaf extracts obtained using HRE –heat reflux extraction (red), UAE – ultrasonic assisted extraction (blue), and ASE – accelerated solvent extraction (green).

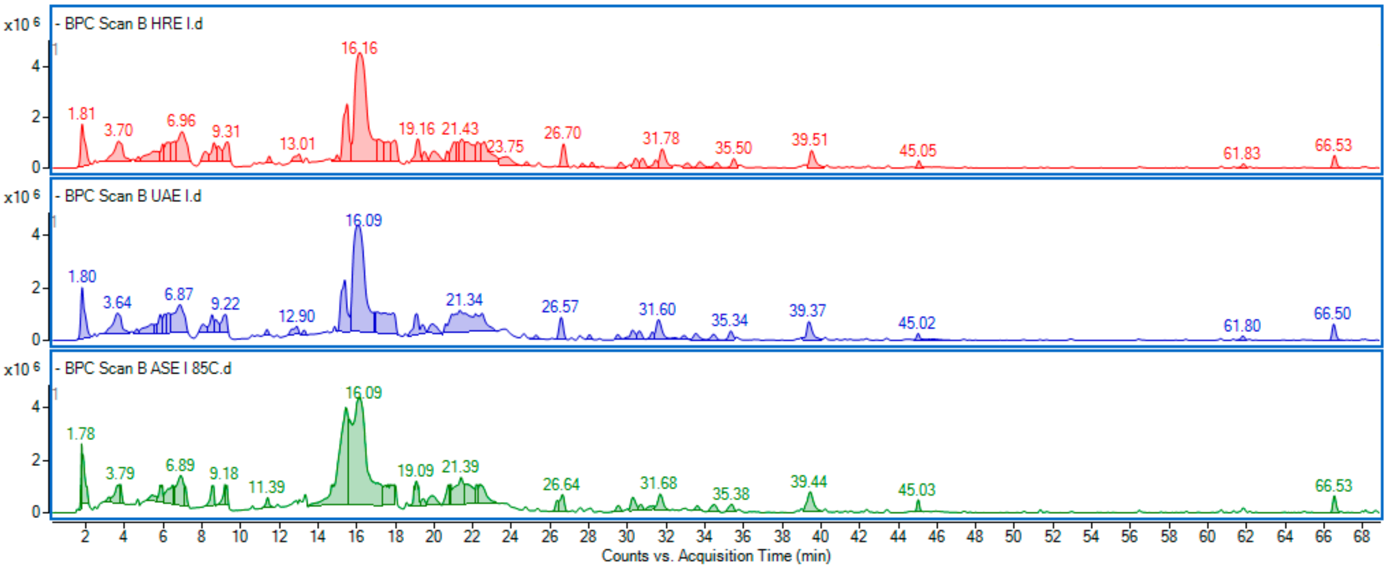

Figure S4. Base ion chromatograms of *H. virginiana* bark extracts obtained using HRE – heat reflux extraction (red), UAE – ultrasonic assisted extraction (blue), and ASE – accelerated solvent extraction (green).
